# Dysregulated biodynamics in metabolic attractor systems precede the emergence of amyotrophic lateral sclerosis

**DOI:** 10.1371/journal.pcbi.1007773

**Published:** 2020-04-15

**Authors:** Paul Curtin, Christine Austin, Austen Curtin, Chris Gennings, Claudia Figueroa-Romero, Kristen A. Mikhail, Tatiana M. Botero, Stephen A. Goutman, Eva L. Feldman, Manish Arora

**Affiliations:** 1 Department of Environmental Medicine and Public Health, Icahn School of Medicine at Mount Sinai, New York, NY, United States of America; 2 Department of Neurology, University of Michigan, Ann Arbor, MI, United States of America; 3 Department of Cariology, Restorative Sciences and Endodontics, School of Dentistry University of Michigan, Ann Arbor, MI, United States of America; University of Connecticut School of Medicine, UNITED STATES

## Abstract

Evolutionarily conserved mechanisms maintain homeostasis of essential elements, and are believed to be highly time-variant. However, current approaches measure elemental biomarkers at a few discrete time-points, ignoring complex higher-order dynamical features. To study dynamical properties of elemental homeostasis, we apply laser ablation inductively-coupled plasma mass spectrometry (LA-ICP-MS) to tooth samples to generate 500 temporally sequential measurements of elemental concentrations from birth to 10 years. We applied dynamical system and Information Theory-based analyses to reveal the longest-known attractor system in mammalian biology underlying the metabolism of nutrient elements, and identify distinct and consistent transitions between stable and unstable states throughout development. Extending these dynamical features to disease prediction, we find that attractor topography of nutrient metabolism is altered in amyotrophic lateral sclerosis (ALS), as early as childhood, suggesting these pathways are involved in disease risk. Mechanistic analysis was undertaken in a transgenic mouse model of ALS, where we find similar marked disruptions in elemental attractor systems as in humans. Our results demonstrate the application of a phenomological analysis of dynamical systems underlying elemental metabolism, and emphasize the utility of these measures in characterizing risk of disease.

## Author summary

The metabolism of essential elements in early life is essential to healthy growth and development. Elemental homeostasis is typically studied by characterizing distributions of elemental concentrations at the level of the population. Here, we introduce a new method of characterizing elemental metabolism at the level of the individual. Using tooth-based biomarkers, we tracked the longitudinal trajectory of essential elements throughout childhood at weekly temporal resolution from birth through approximately 10 years of life. We analyzed these trajectories to identify the formation of stable dynamic states (attractors) and transitions between these states throughout development. We found that metabolic dynamics were specific to discrete elemental pathways; copper metabolism typically involved the formation of multiple discrete states throughout childhood, whereas other elements, such as zinc, tended to persist in a single stable dynamic throughout development. Next, we compared elemental biodynamics in neurologically healthy cases and subjects that were later diagnosed with amyotrophic lateral sclerosis (ALS). We found these patterns were dysregulated in ALS, and also found similar results in a mouse model of ALS. Overall, our results provide a novel approach to characterize elemental biodynamics throughout development, and emphasize that the dysregulation of these processes may be predictive of later onset of disease.

## Introduction

Biological systems are highly dynamic, exhibiting time-dependent patterns that develop in early-life and organize into recurrent processes ranging from milliseconds as observed in neurons firing [1–3] to months as in the menstrual cycle, or longer as in pregnancy [4–7]. However, much of our knowledge of how human physiology integrates environmental exposures is based on single time-point measures of elements and molecules, which neither captures time-dependent dynamical states, nor tracks longitudinal state transitions, which may be characteristic or predictive of emergent phenotypes.

Two essential challenges have prevented the characterization of dynamical systems involved in human development over time-scales spanning childhood through early adult maturation. First, the capacity to track fine scale temporal changes in human systems requires the ability to take hundreds of serial *in vivo* measurements of biological processes over the span of childhood when organ systems and networks are rapidly developing, which is precluded by typical methods of biomarker assessment in blood or urinary samples. Second, even when the capacity to capture longitudinal biomarker profiles is available, the underlying dynamics of these systems remain largely unexplored; it is thus uncertain if these systems should be studied through the lens of chaotic, deterministic, or stochastic dynamics. Here, we demonstrate an approach to overcome these barriers through the combination of novel analytical technologies and computational tools.

We used fine scale temporal profiles from novel teeth biomarkers to track the uptake of nutrient elements and non-essential metals throughout childhood. We used permanent teeth of adult participants to obtain weekly retrospective measures from birth to adolescence using our well-validated approach [8]. With this method, teeth are analyzed along their growth increments (akin to growth rings in trees) using laser ablation-inductively coupled plasma-mass spectrometry. This analysis provides longitudinal profiles of metals over the ages the tooth dentine is developing.

Given the uncertain nature of the processes underlying elemental homeostasis, we developed two distinct analytical approaches, each based on fundamentally differing assumptions, to characterize dynamical systems involved in elemental metabolism at the level of the individual. First, to characterize the complexity of metabolic state formation throughout development, we analyzed the potential energy landscape of longitudinal exposure profiles to identify discrete quasi-stable metabolic states. This approach, based on the assumption of an underlying stochastic process, allowed the characterization of discrete attractor systems, reflecting a set of numerical values toward which a system develops [9–14]. These are interpreted as quasi-stable states in elemental homeostasis that emerge throughout development. In parallel, we also applied an analytical approach focused on quantifying underlying periodicity in elemental assimilation through the application of recurrence quantification analysis (RQA). In combining measures derived from each of these approaches, we can thus characterize complexity and stability in state formation and periodicity in the metabolism of essential and non-essential elements throughout development. In essence, we used these different methods because we do not make an a priori assumption that the systems we are studying are reliably chaotic, deterministic, or stochastic.

To test the fundamental relevance of these childhood systems to life-long human health trajectories, we applied these methods to samples from participants with a well-known neurological disease, amyotrophic lateral sclerosis (ALS), a progressive and fatal neurodegenerative disorder affecting motor neurons [15]. We choose ALS as an exemplar disease because exposures to metals and elements are identified risk factors, and it is known that environmental factors can influence disease risk and disease survival [16–18]. It remains unknown whether causative environmental perturbations, including exposure to metals, may occur during early-life in future ALS patients. Because of the low incidence rate of ALS and its clinical emergence in late adulthood, prospective studies would require large birth cohorts to be maintained for decades, which hampers research on early life determinants of ALS risk. Here, we reconstructed exposures in the first 10 years of life from permanent teeth of ALS participants and healthy controls, and applied dynamical analytical methods to characterize the emergence of quasi-stable states and periodicity in elemental metabolism. We subsequently leveraged these indicators in the implementation of comparative and predictive statistical models to understand how these factors relate to ALS. Our findings highlight the importance of understanding homeostatic dynamics involved in elemental assimilation during early life as risk factors for later-life neurodegenerative disorders.

## Results

### Attractor landscapes in childhood elemental metabolism

**Fig 1:**
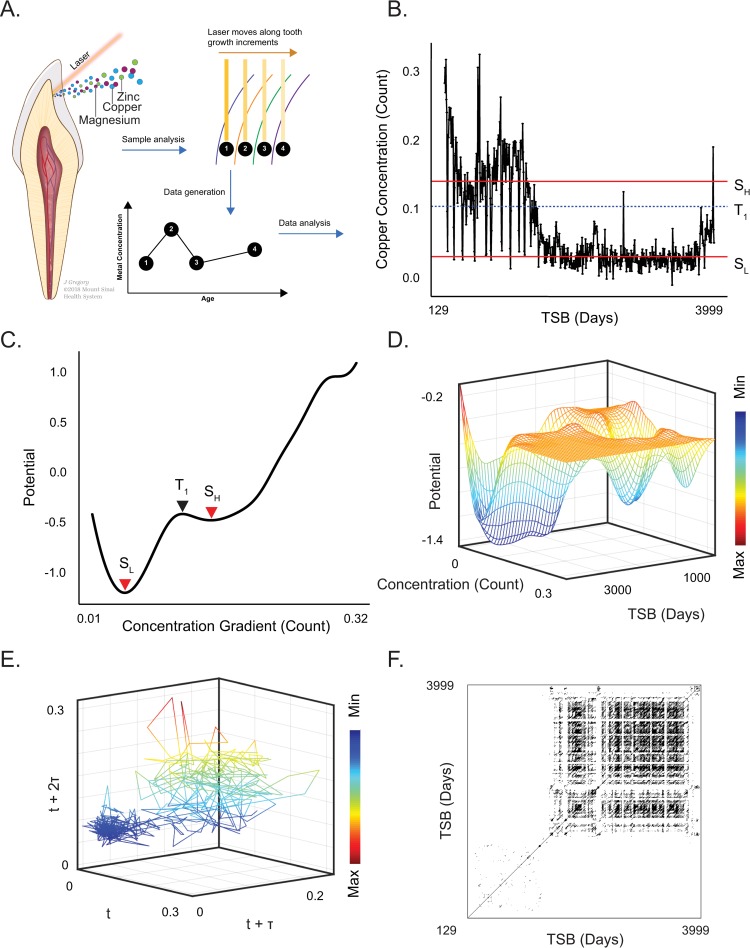
Reconstruction of dynamical processes underlying childhood elemental metabolism. **(A)** Schematic of laser ablation inductively-coupled plasma mass spectrometry (LA-ICP-MS) analysis to measure metal concentrations in dentine growth increments of adult teeth. Elements are simultaneously sampled along sequential growth lines, allowing the temporal concentration profile of each element to be reconstructed over the course of development. **(B)** Example of a copper profile showing calcium normalized counts sampled from 129 days to approximately 10.95 years in an individual control sample. Red lines indicate concentrations associated with the quasi-stable equilibria of a high-concentration, slow-oscillating state (S_H_), and a low-concentration, fast oscillating state (S_L_); the dashed blue line shows the unstable threshold point between states. **(C)** Potential energy profile derived from copper concentrations in (B), with (unitless) measure of potential on the y-axis and copper concentrations on the x-axis. Red arrows specify the local potential minima (and associated copper concentrations) of basins of attraction in potential energy profiles, which indicate formation of quasi-stable attractor states, while the black arrow indicates the transitional ‘tipping point’ between basins of attraction, defined as a local maxima. The number of quasi-stable states observed in each element were measured in each individual then combined for subsequent statistical analysis. **(D)** Three-dimensional contours of potential energies shown in (C) plotted against copper concentrations (as normalized ion counts) and developmental time since birth (TSB). Discrete attractor states appear as localized wells in the potential energy landscape throughout the course of development. **(E)** Phase portrait derived from Takens embedding of copper concentration profile shown in (B). Axes are derived from lag-embedding of copper measurements and are therefore in units of count. Consistent with the potential energy landscape shown in (C, D), this indicates a two-state attractor system. This system is focused toward a broader orbital trajectory at higher elemental concentrations (upper right), or a tight cluster at lower concentrations (lower left). **(F)** Recurrence plot derived from (E) emphasizing a shift from an initial period of slow-oscillating dynamics to a fast-oscillating laminar dynamic. Features derived from recurrence plots, including Determinism, Mean Diagonal Length, and Entropy, were measured in each individual separately then combined for subsequent statistical analysis.

Our tooth matrix biomarkers generate over 500 time-series measurements of essential and non-essential elements covering an average of 10.3 years of childhood and adolescence (Fig 1A and Methods). This method relies on using laser ablation to sequentially sample the growth layers of human tooth dentine and to characterize the elemental composition of the ablated material using mass spectrometry. In this manner we generated temporally longitudinal profiles of elemental uptake during childhood and adolescence [19,20]. Given the uncertain nature of the metabolic processes governing these systems, which could alternatively or successively involve stochastic, deterministic, or chaotic dynamics, we applied two distinct analytical strategies to characterize underlying attractor dynamics from a purely phenomenological perspective. As applied here, attractors represent the emergence of quasi-stable state dynamics during the establishment and development of biological processes [9,21–23].

First, to characterize the emergence of quasi-stable metabolic states during childhood and adolescence, we implemented a potential energy analysis to identify basins of attraction underlying changes in elemental concentration. To illustrate this process, Fig 1B shows raw copper measurements in one neurologically normal control subject, capturing concentrations from 129 days after birth to approximately 10.95 years of age. In Fig 1C, we show the potential energy profile derived from this trace, with the gradient of observed concentrations on the x-axis against the (dimensionless) potential energy associated with varying copper concentrations on the y-axis. Quasi-stable attractor states, referred to as basins of attraction, are detected as local minima in the potential energy function, as indicated in Fig 1C by S_L_ and S_H_, referring to low- and high-concentration states, respectively. Separating these, at point T_1_, the local maxima in potential energy identifies the threshold between states. In Fig 1B, the concentrations corresponding to S_L_, S_H_, and T_1_ are identified with red or dashed blue lines, respectively, to emphasize how the system transitions between states throughout development.

This example (Fig 1B) illustrates that copper metabolism transitions throughout childhood between a high-concentration and slow oscillating state (S_H_, above T_1_), followed by a low-concentration and fast-oscillating state (S_L_, below T_1_). The relatively shallow attractor basin (Fig 1C) associated with the high-concentration state, S_H_, indicates that this state is relatively unstable and easily perturbed; consistent with this, we observed that even at times when this state is predominant, the system repeatedly transitions to the alternative state and back again. The stability of the low-concentration state, S_L_, in contrast, is comparatively robust, as reflected in the depth of its corresponding basin of attraction (Fig 1C, S_L_), though the system continues to transition between these states to some extent throughout development. In Fig 1D, we visualize these dynamics through the estimation of potential energy in rolling windows, which allows the potential energy contours to be simultaneously visualized as a function of concentrations and developmental timing. As in the two-dimensional potential energy profiles, attractor basins in this visualization, apparent as momentary wells or persistent troughs, signify quasi-stable attractor basins indicative of homeostatic stability in elemental metabolism.

While the analysis of potential energy landscapes allows the quantitation and visualization of quasi-stable state formation, this approach is insensitive to periodic aspects of attractor dynamics. To capture these, we also pursued attractor reconstruction by phase space embedding of elemental profiles using Takens embedding theorem [24], as in Fig 1E, and subsequent recurrence quantification analysis (Fig 1F). Consistent with the indicators of bistability derived from the potential analysis in this example, attractor reconstruction via Takens embedding yields two distinct orbital clusters in phase space, which transition between a tight cluster associated with lower concentrations (lower left of Fig 1E) and a broader orbital trajectory associated with higher concentrations (the upper right of Fig 1E). Finally, we applied recurrence quantification analysis (RQA) to quantify the prevalence (determinism), duration (mean diagonal length), and complexity (Shannon entropy) of periodicity in these systems. Consistent with the potential energy landscapes and the Takens embedding, the resulting recurrence plot shown in Fig 1F emphasizes a developmental shift from an initial period of slow-oscillating dynamics that ultimately shift to a fast-oscillating laminar dynamic.

**Fig 2:**
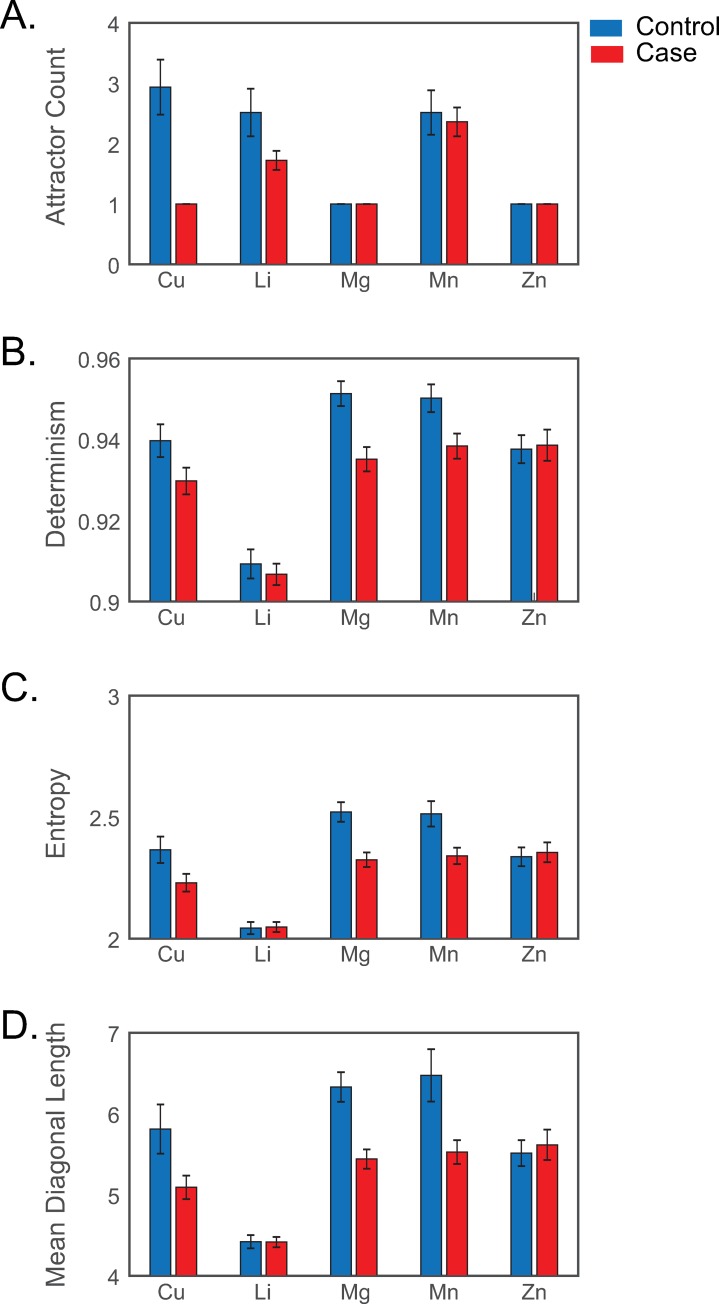
Distribution of elemental attractor states and recurrence features in controls and ALS cases. In **(A)**, the mean (± SEM) number of quasi-stable states identified in healthy controls (blue bars) and ALS cases (red bars) are shown in varying elemental pathways. (**B, C, D**) show RQA-derived mean ± SEM Determinism (B), Entropy (C), or Mean Diagonal Length (D) for ALS cases (red bars) and controls (blue). Abbreviations: Copper (Cu), Lithium (Li), Magnesium (Mg), Manganese (Mn), Zinc (Zn).

In combining these approaches, we characterized the dynamics underlying these systems from two distinct phenomological perspectives, focused either on the emergence of discrete homeostatic states, as derived from potential analysis, or the measurement of periodic prevalence, duration, and complexity, as derived from RQA. Complementing these four measurements, the visualization of potential energy landscapes, Takens embeddings, and/or recurrence plots can be used to evaluate consistencies across methodologies, and dissect time-varying dependencies; for example, the persistence of states over time, and transitions between them. We extended these approaches to other metals and, similar to copper, found that lithium and manganese exhibited 2–3 quasi-stable states over childhood and adolescence. Examples of these are shown in S1 Appendix Figures A and B, and the distribution of quasi-stable states and recurrence features (determinism, mean diagonal length, entropy) observed across different elemental pathways are shown in Fig 2 and detailed in S1 Appendix Table A. By contrast, other elements, such as magnesium and zinc, only showed a single attractor state that persisted throughout development.

### Attractor dynamics and periodicity of elemental metabolism are perturbed in ALS

To demonstrate the relevance of these features to human health and development in a practical case example, we undertook a comparison of attractor dynamics characterized in 36 ALS and 31 control participants (participant characteristics are shown in S1 Appendix Table B). In five elemental pathways, including copper, lithium, magnesium, manganese, and zinc, we used potential energy analysis to measure the frequency of stable state formation, and used recurrence quantification analysis (RQA) to characterize periodic dynamics, quantified in measures of determinism (cycle prevalence), mean diagonal length (MDL; cycle duration), and Shannon entropy (cycle complexity). Measuring these features, per element, in each individual subject allowed the characterization of dynamics at the level of the individual, but also provided measures useful for testing practical statistical hypotheses at the level of comparing cases vs. controls. The distributions of these features in cases and controls are shown in Fig 2. For each of these features, we tested the hypothesis that ALS cases differed from controls in elemental attractor dynamics during childhood, decades before the clinical signs of neurodegeneration are evident.

**Fig 3:**
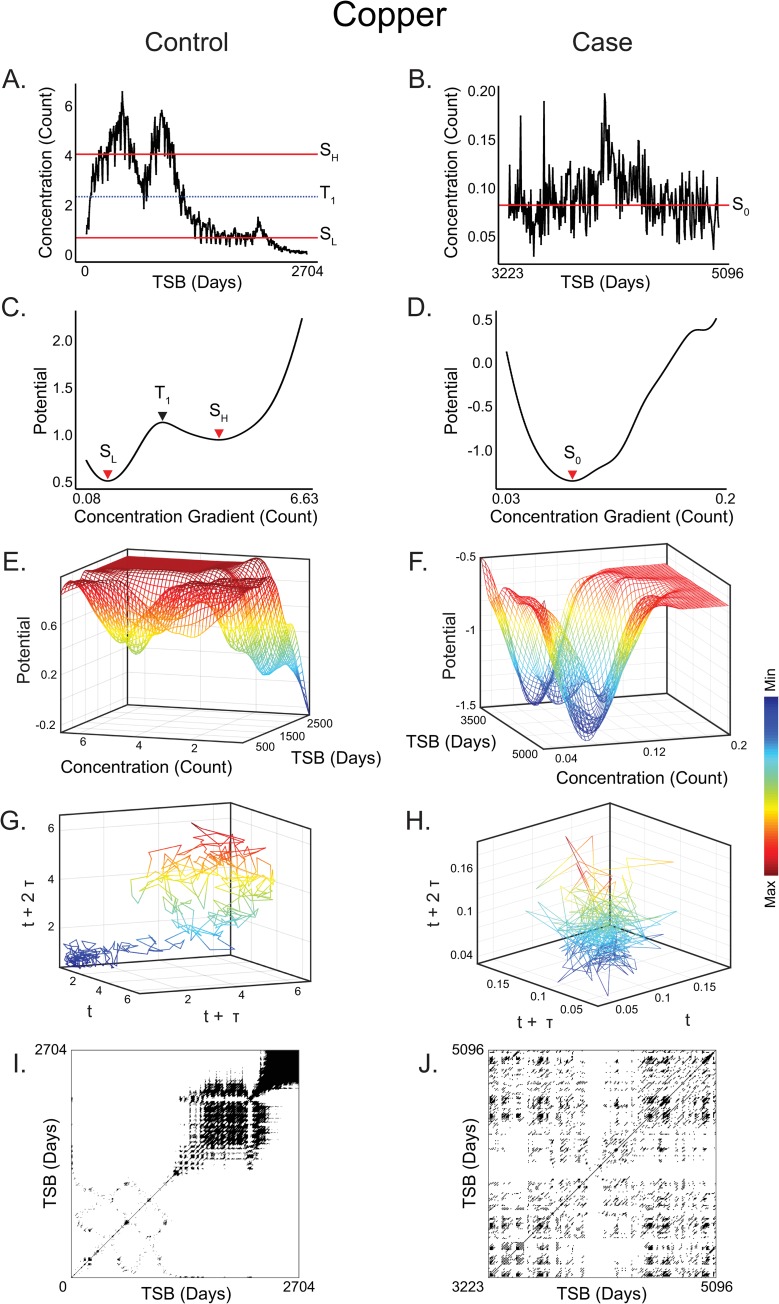
Copper attractor dynamics and metabolic periodicity in controls and ALS cases. **(A)** Copper concentration profile over developmental time in a healthy control. Red lines indicate equilibria for high (S_H_) and low (S_L_) concentration quasi-stable states, as identified in local minima of the potential analysis in (B), and blue dashed line indicates the unstable equilibria between these states. **(B)** Copper concentration in an ALS case. Red line indicates the single quasi-stable state identified from potential analysis in this system. **(C)** Control copper potential energy profiles corresponding to concentrations in (A), showing two basins of attraction (red arrows; S_L_ and S_H_), and the transition point between them. These are identified as the local minima and maxima, respectively, of the potential energy function. **(D)** In contrast to (C), the ALS case corresponding to (B) conforms to a single attractor system. **(E)** Three-dimensional contours of potential energies plotted against copper concentrations (as calcium normalized ion counts) and developmental time since birth in a healthy control. Discrete attractor states appear as localized wells in the potential energy landscape throughout the course of development. **(F)** Unlike the bistable system shown in (E), in the ALS case copper metabolism is dominated by a single attractor system that emerges early and persists throughout development. Plateau indicates that any departure from the attractor well was highly transient. **(G)** Phase portrait derived from a healthy control subject, again suggesting the formation of a bistable attractor system, focused either toward a tight cluster in the lower left, or a broader orbital trajectory toward the upper right. Axes are derived from lag-embedding of copper measurements and are therefore in units of count. **(H)** Phase portrait of copper concentration profile from an ALS case, indicative of a singular stable state throughout development. **(I)** Recurrence plot of healthy control derived from (G) emphasizing a shift from an initial period of slow-oscillating dynamics to a fast-oscillating laminar dynamic. **(J)** Recurrence plot of ALS case derived from (H) showing disruption and loss of structure. Time axis extends from birth to a maximum of 13.96 years.

The distribution of state measurements for cases and controls is provided in Fig 2A and S1 Appendix Table A. We found that copper metabolism typically presented as a multi-state attractor system in control subjects (Fig 3A, 3C and 3E), but in 100% of ALS cases copper regulation was dominated by a single attractor yielding a mono-stable system that persisted throughout development (Fig 3B, 3D and 3F). Phase portraits derived from Takens embedding showed patterns consistent with indicators of bi-stable/multi-stable (controls) or mono-stable (ALS cases) systems. Examples shown in Fig 3 illustrate, in a typical control subject, a tight orbital cluster at low concentrations (lower left of Fig 3G) that transitions towards a broader orbital trajectory at higher concentrations (upper right of Fig 3G); whereas, similar analyses in an ALS case (Fig 3H) suggest a singular state. Statistical analyses confirmed the reduction in stable state formation in ALS cases was significant for copper (raw *P* < 0.001; FDR-adjusted *P* < 0.001). We observed a similar trend in lithium (raw *P* = 0.03; FDR-adjusted *P* = 0.07; see S1 Appendix, Figure A, panels A-D) metabolism, whereas the number of stable states formed in other elements, for example manganese (see S1 Appendix, Figure A, panels E-H) was unperturbed in ALS. Additional details on statistical analyses are provided in S1 Appendix Table C.

Beyond analyzing the stability of metabolic attractor systems, we also applied RQA to characterize the periodicity of elemental metabolism. We found that determinism, entropy, and mean diagonal length (MDL), indicative of cycle prevalence, complexity, and duration, respectively, were significantly dysregulated in multiple attractor systems (Fig 2B–2D, S1 Appendix Table D). In the case of copper, a recurrence plot derived from a typical example showed two distinct regions, an initial period of slow-oscillating dynamics (lower left of Fig 3I) with a sharp transition to a fast-oscillating laminar dynamic (upper right of Fig 3I). In an ALS case, by contrast, this periodic structure in copper metabolism was lost (Fig 3J). Statistical tests of RQA features for copper and other metals are detailed in S1 Appendix Table D; notably, we also observed significant dysregulation of periodic dynamics in magnesium and manganese pathways.

### Attractor dynamics and periodicity of elemental metabolism in animal models

In wild-type mice, we examined if elemental metabolism would organize itself into high-dimensional attractor systems similar to what was observed in our human controls. Given the highly regulated environment of laboratory mice, presence of stable attractor basins demarcated from unstable regions by tipping points would provide additional evidence that attractor dynamics were a physiologic norm of elemental metabolism and not solely due to fluctuations in environmental exposures. As in control humans, we found that in wild-type mice, attractor dynamics differed across elemental pathways, with some elements, such as magnesium, exhibiting multiple attractor systems in control mice, whereas others, such as manganese, only showed a single attractor basin (S1 Appendix Figures C and D, Table E).

**Fig 4:**
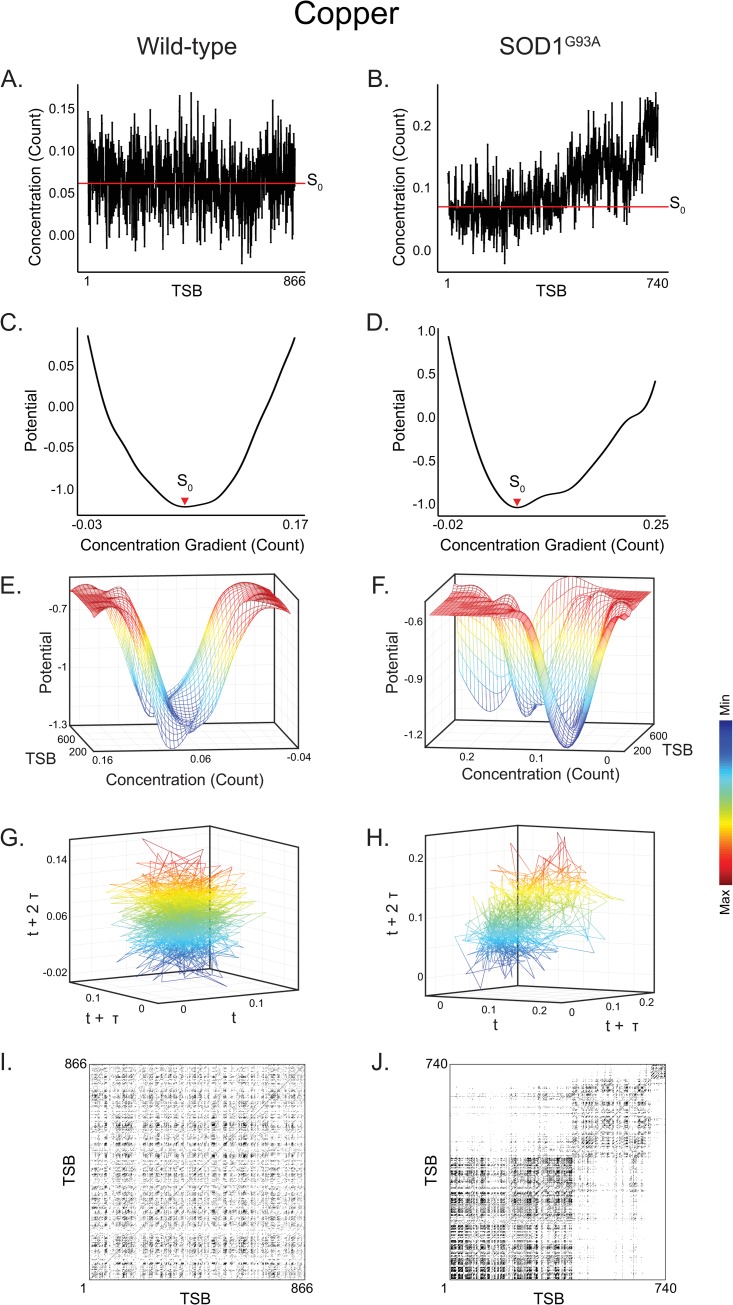
Copper dynamical attractor systems in an animal model of ALS. **(A)** Copper concentration in a wild-type mouse. Red line indicates singular quasi-stable state identified from potential analysis in (C). **(B)** Copper concentration in a SOD1^G93A^ mice. Red line indicates singular quasi-stable state identified from potential analysis in (D). **(C)** Wild-type copper potential energy profiles corresponding to concentrations in (A). Red arrow (S_0_) indicates singular local minima identified in potential energy function, indicative of a single basin of attraction. **(D)** SOD1^G93A^ potential energy profiles corresponding to concentrations in (B). Red arrow (S_0_) indicates singular local minima identified in potential energy function, indicative of a single basin of attraction. **(E)** Three-dimensional contours of potential energies plotted against copper concentrations and developmental time in a wild-type mouse and **(F)** SOD1^G93A^. **(G)** Phase portrait derived from (E). Axes are derived from lag-embedding of copper measurements and are therefore in units of count **(H)** Phase portrait derived from (F). **(I)** Recurrence plot of wild-type mouse derived form (G). **(J)** Recurrence plot of SOD1^G93A^ derived from (H) emphasizing the emergence of periodic structure.

Due to the unknown genetic etiology of sporadic ALS, there are no transgenic animal models that correspond exactly to the human condition; however, we used a mouse model of familial ALS overexpressing mutant superoxide dismutase-1 (SOD1) with a guanine to alanine point mutation at amino acid 93, which exhibits neurodegeneration similar to human ALS [25,26]. We compared the elemental attractor patterns in control mice to those observed in SOD1^G93A^ mice to determine if a genetically induced alteration in metal-dependent pathways would be apparent in the dynamics of elemental metabolism. Similar to our human data, we observed marked differences in the periodicity of copper attractor systems between WT and SOD1^G93A^ mice, in that we identified significant differences in copper determinism (raw *P* = 0.0008; FDR-adjusted *P* = 0.01) (Fig 4 and S1 Appendix Table F). As well, we noted differences in Li dynamics, though these did not survive adjustment for multiple comparison (see S1 Appendix Table F). Contrary to our observations in humans, in the animal model we found no alterations in the stability of attractor systems (assessed via the frequency of attractor basins; see S1 Appendix Table E). Fig 4 shows example data emphasizing these general trends in copper metabolism derived from WT and SOD1^G93A^ mice. In Panels C-F, potential energy landscapes derived from the WT and ALS animal model exhibit a similar pattern of persistent metabolic state formation, while the Takens embedding and recurrence analysis (Panels G-J), emphasize the dysregulation of periodic dynamics. In SOD1^G93A^ mice ALS was associated with elevated determinism in copper metabolism, whereas copper determinism was reduced in humans. Although no direct comparison of the transgenic mouse data with sporadic human ALS cases is possible, an important finding was the dysregulation of copper metabolism in both scenarios.

### Elemental attractor dynamics as an early warning system for ALS

Given the divergence of attractor dynamics in ALS cases *vs* controls decades before the emergence of symptoms, we next tested the predictive utility of these measures in classifying ALS. In essence, we explored if there are elemental signatures present during childhood development that distinguish controls from individuals who will later develop ALS in adulthood. Using features derived from potential analysis and RQA, which included the number of quasi-stable states and RQA-derived determinism, MDL, and entropy measured in each element for every subject, we implemented two classification algorithms to determine if early-life elemental dynamics were predictive of ALS.

**Fig 5:**
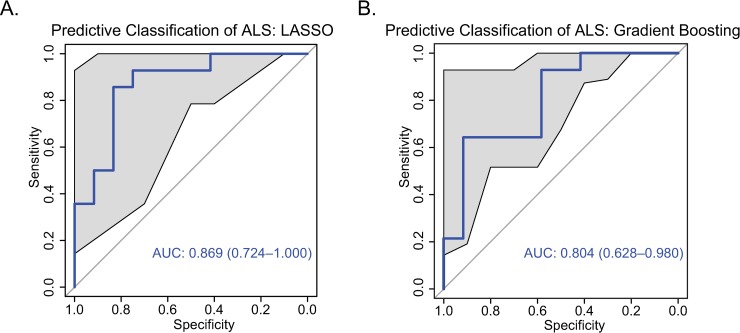
ROC curve for predictive models classifying ALS cases and controls. **(A)** Lasso model. **(B)** Tree-based gradient boosting classifier. Shaded area shows confidence interval of the sensitivity at varying specificities estimated across 2000 bootstrapped permutations of predicted outcomes; AUC shows estimated mean area-under-curve and 95% CI intervals.

In our initial approach, we implemented a penalized logistic classification algorithm (LASSO) to train a model predictive of ALS on 60% of the total dataset. When we applied this to a testing set (40% of the data), with an optimal decision making threshold of 0.527 defined by maximizing the distance from the central diagonal, this model was 84.6% accurate in predicting case/control status. To quantify uncertainty in model predictions, we bootstrapped (N = 2000) predicted outcomes to estimate error in our ROC curve (Fig 5A), yielding an estimated AUC of 0.87 (0.72–1.00), which we confirmed differed from performance expected at chance (*P* = 0.0004). S1 Appendix Table G shows effect estimates for each feature selected in this model, with RQA-derived metrics of copper, magnesium, manganese, zinc, and lithium contributing most to this prediction. To confirm the predictive efficacy of these top features, we conducted a follow-up analysis utilizing only the top 5 features, defined as those with highest (absolute value) effect estimates. This model performed comparably to the original model constructed on the full feature set, with AUC of 0.85 (0.68–1.00).

We followed this analysis with the parallel implementation of a tree-based classifier to confirm the predictive efficacy of these features was robustly generalizable. As in the implementation of the LASSO algorithm, the available data were divided into training (60%) and testing (40%) sets, with predictive efficacy ultimately evaluated in the testing set. We found that predictive efficacy was generally similar in the tree-based classifier, yielding an AUC of 0.8 (0.62–0.98), as shown in Fig 5B. As with the LASSO, we found that model performance significantly exceeded chance (P = 0.001). The features that contributed most to model performance are identified in S1 Appendix Table H; as in the LASSO model, these included RQA-derived metrics of copper, magnesium, and manganese performance. Testing predictive efficacy in a model including only the 5 most-predictive features yielded a predictive model with an AUC of 0.76 (0.57–0.94).

## Discussion

We demonstrate a novel approach to probe the dynamics underlying elemental assimilation and metabolism, and how these processes are associated with health and disease. To achieve this, we combined methodologies in exposure biology, dynamical systems theory, and biostatistics. First, at the level of biomarker assessment, we developed tooth biomarkers that provide over 500 consecutive time-series measures of elemental metabolism from birth to approximately 15 years of age, with an average time span in our cohort of 10.3 years. To characterize dynamics involved in elemental assimilation, we applied methods derived from dynamical systems theory to measure periodicity and quasi-stable state transitions throughout development. These phenomological indicators, derived at the level of the individual, provided the basis for a subsequent statistical analysis to test for differences in these processes between healthy subjects and ALS cases, and to develop algorithms predictive of disease. Our results identify significant differences in these features between ALS cases and controls, which we subsequently extended to an SOD1 ALS mouse model system.

The rationale for a phenomological analysis of system dynamics is to provide a new methodological perspective on the analysis of elemental assimilation and metabolism during childhood development. Whereas elemental homeostasis is typically studied through the establishment of population distributions, and an individuals’ health assessed through their momentary concentration relevant to this distribution, here we demonstrate the utility of characterizing homeostatic equilibria at the level of the individual, as measured through the formation of quasi-stable states and cyclical dynamics. The measures derived from the analyses we present provide a new avenue for epidemiological studies that traditionally employ statistical hypothesis testing seeking to detect concentration differences between health and disease. Future efforts will build on this work by investigating the connection between state transitions and anthropometric indicators of growth and development, and/or explore links to disease.

The foremost challenge in studying a previously-uncharacterized and therefore interesting biological system is that we do not know (or it is not possible to know) whether the observed time series data reflects a chaotic, deterministic, or a stochastic process, or if the system transitions between these states. These uncertainties present a challenge in determining the appropriate assumptions under which to analyze the system. To overcome this, we applied parallel and complementary dynamical methods which are sensitive to varying aspects of the underlying attractor system. Each of these approaches is useful in quantifying dynamics that the alternative method may be insensitive to; RQA, for example, provides direct measures of the prevalence (determinism), duration (mean diagonal length, MDL), and complexity (entropy) of cyclical processes, but does not directly quantify state shifts (though indicators of these may be observed in Takens embedding and recurrence plots). In contrast, potential analysis is useful in quantifying the emergence of discrete attractor states, but does not provide direct indicators of periodicity in these systems. By combining these approaches, it is thus possible to capture phenomological dynamics that might be missed by either analysis applied alone, and would be ignored entirely in standard approaches to biomarker assessment.

To examine whether these approaches are of value in detecting disease signatures, we focused on ALS, a late-onset neurodegenerative disease believed to be triggered by early-life environmental factors acting in conjunction with genetic predisposition [27,28]. In individuals with ALS, we observed disruptions in the architecture of elemental attractor systems, evident in the dysregulation of bistable systems, which in ALS subjects typically presented as singular attractor states. For example, while copper and lithium pathways showed multiple attractor states in healthy control individuals, ALS was associated with the formation of fewer attractor states, typically yielding a single attractor that persisted throughout development. We characterize the emergence of multiple attractor states as an indicator of bistability because these systems appear to freely transition between states, which is in contrast to behavior indicative of a bifurcation, wherein departure from a given state implies its sublimation. In a few human cases, and in some mice, we further observed the formation of multi-stable states, where three or more quasi-stable states were observed.

The dysregulation of attractor dynamics in ALS when compared to controls was additionally apparent in our analysis of periodicity via Takens embeddings and RQA. Consistent with our analysis of potential energy landscapes, we observed significant dysregulation in the prevalence, complexity, and duration of periodic processes in elemental metabolism, as quantified in determinism, entropy, and mean diagonal length (MDL). This pattern was evident in copper, magnesium, and manganese pathways, though not in zinc and lithium. These results emphasize that varying dynamical methods may capture different aspects of attractor dysregulation. Further, the observed early-life dysregulation of periodicity in elemental metabolism is consistent with recent findings which relate to the emergence of autism spectrum disorder (ASD) [29], though that study focused on prenatal and perinatal elemental dynamics. Nonetheless these studies together emphasize the importance of assessing elemental metabolism for neurological disorders.

Considered together, the findings in our human participants suggest that ALS pathology involves broad dysregulation of various mechanisms that regulate metabolism of copper, and specific aspects of the metabolism of other elements including manganese, lithium and magnesium. Supporting this, both linear and tree-based classification algorithms that were used for the predictive analysis identified these elements as the most important contributors to predictive performance; and, follow-up analyses which utilized only features derived from these elements yielded good performance. Our data are in agreement with clinical and experimental studies revealing copper dyshomeostasis in ALS [30], and add to the growing literature relating lithium regulation to ALS [31,32].

We further extended our findings in human subjects to a mouse model of ALS expressing the human G93A mutant SOD1 protein, which results in neurodegeneration and a phenotype similar to human familial ALS. As in healthy humans, in wild-type mice we also observed the formation of multi-state attractor dynamics in developmental elemental assimilation. Consistent with our findings in humans, the transgenic mice showed dysregulated copper and lithium metabolism, primarily captured in the RQA-based measures of periodicity in these pathways. As shown in (Fig 4G and 4H), we did not observe differences in quasi-stable state formation in the animal model as we did in humans. While the transgenic mouse model is not a direct correlate of sporadic ALS (sALS) cases, it allowed us to confirm that genetically induced metabolic perturbations are expressed as disrupted attractor dynamics of essential elements. As in our analysis of human subjects, however, these findings should nonetheless be interpreted from the perspective of an observational study; that is, while the underlying genetics in the mouse model were experimentally controlled, we did not directly manipulate the elemental dynamics, and should therefore interpret the observed changes in these systems as associative.

Collectively, our data show that both the stability and plasticity of elemental metabolic attractors, and the systematic consolidation of metabolic pathways, relate to important indices of normal development. We also discovered previously uncharacterized aspects of metabolic pathways during neurodevelopment that related to the eventual onset of ALS. It is important to consider our results in the context of the known environmental and genetic risk factors for ALS. Aberrant levels of essential and toxic metals are found in biospecimens from ALS patients. Some of those metals have been associated with biological pathways altered in ALS, such as oxidative stress and mitochondrial dysfunction [33]. Over 25 genes have been identified either as the genetic cause of the disease in the familial cases, or as genetic variants influencing predisposition in sALS, the most common form of the disease [34,35]. Proteins encoded by some of these genes require manganese, copper, and zinc as cofactors for their enzymatic activity, or are interrelated to other elemental pathways [36]. Previous studies have largely relied on elemental biomarkers or surrogate estimates of exposure at a few time points after diagnosis [34]. Our data show that elemental metabolic pathways are complex and vary over time. Our approach, therefore, offers an alternative to explore gene-environment interactions by including measures of attractor landscapes underlying elemental metabolism. Our study is limited by a small sample of participants and was undertaken at one clinical center, which limits generalizability; as well, since our sampling method is dependent on the quality of the tooth sample, we were unable to sample exactly equivalent developmental windows in all participants. We used self-reported measures of smoking but did not have biomarkers of tobacco smoke exposure.

Overall, we show the existence of elemental attractor landscapes spanning approximately a decade of childhood. The utility of these features in predictively classifying ALS and other disorders, from measures derived decades before the emergence of clinical signs and symptoms, emphasizes both the theoretical and practical significance of developing sentinel approaches that apply dynamical systems methods to biological samples.

## Methods

### Study participants

ALS participants, meeting revised EI Escorial Word Federation of Neurology criteria [37] (N = 36) were recruited at the University of Michigan ALS clinic (S1 Appendix Table B). Clinical and family history data were obtained. Age- and sex-matched control participants undergoing multiple teeth extractions were recruited at the Oral Surgery Clinic at the University of Michigan Dental School. Control subjects (N = 31) were excluded if they or a first- or second-degree family member had a neurodegenerative disease. Participants or next of kin provided informed consent. This research was approved by the University of Michigan Medical School Institutional Review Board. Permanent teeth obtained at autopsy or during routine dental extractions were gently cleaned to remove tissue, rinsed with deionized water, air dried, and stored at room temperature, as metal deposits in teeth are known to be stable and reflect exposure during development [19].

### Animal model of ALS and sample collection

Fourteen (7 male and 7 female) B6.Cg-Tg(SOD1*G93A)1Gur/J (SOD1^G93A^) and 15 (8 male and 7 female) control littermate mice (JAX stock #004435; Jackson Laboratories, Bar Harbor, Maine) were maintained with a Lab Diet 5LOD chow (Lab Diet, St. Louis, MO) and housed at the University of Michigan following the Committee on the Care and Use of Animals guidelines (approval #PRO00008431) [38]. Animals were sacrificed at the age of 41 d, 73 d, 120 d, and end stage using pentobarbital (Vortech, Dearborn, MI). End stage (161–175 d) was determined based on the health of the mouse. An age and gender matched control was sacrificed at the same time point for each SOD1^G93A^ mouse. Genotyping was performed by PCR as suggested by supplier (Jackson Laboratories). The lower mandibles were dissected and the teeth removed for metals analysis.

### Metals analysis of teeth

Metal concentrations in deciduous and permanent teeth have been validated against levels in environmental samples and in blood in both human and animal studies [19,20,39–41]. Our method is detailed in S1 Appendix Procedures and Figures E and F. Metal concentrations were determined by laser ablation inductively-coupled plasma mass spectrometry (LA-ICP-MS) [42–44]. Briefly, we used a New Wave Research NWR-193 (ESI, USA) laser ablation unit equipped with a 193 nm ArF excimer laser connected to an Agilent Technologies 8800 triple-quad ICP-MS (Agilent Technologies). The laser was scanned in dentine from the dentine horn tip towards the tooth root tip and surface contamination was removed using a pre-ablation scan. Data were analyzed as metal (metal studied) to calcium (internal standard) ratios to control for any variations in mineral content within a tooth and between samples. Each tooth was sampled, on average, at over 500 locations. The method and operating parameters for LA-ICP-MS are shown in S1 Appendix Procedures and Table I. For the animal model, identical instrument operating conditions were applied. Concentration of multiple metals of 14 mice was determined in the incisor dentin by LA-ICP-MS. The majority of samples were first molars (43%), followed by second molars (24%), canines (13%), incisors (12%), premolars (4%), and third molars (3%). Statistical analysis accounted for tooth type in the analysis. Temporal information was assigned using well-established ages for the initiation and termination of tooth development (for review see: [45]).

### Phase space reconstruction

For many dynamical methods, including those applied here, the reconstruction of time series data in a phase space coordinate system via Takens embedding is an initial step in evaluating orbital trajectories, recurrence parameters, and attractor topology. We used delay embedding for phase space reconstruction, [24] which involves the specification of delay parameter, *τ*, and embedding dimension, *m*, to reconstruct an *m*-dimensional coordinate system from a univariate time series, in this case an elemental measurement from a single subject. To estimate the appropriate delay parameter (*τ*), a mutual information algorithm was applied, with the given delay corresponding to the minimal interval needed to minimize mutual information [46,47]. Similarly, the minimization of a false nearest neighbors (FNN) algorithm determined the appropriate embedding dimension, *m*. For each elemental pathway, per person, these parameters were derived as described above for use in phase space reconstruction and subsequent analysis with recurrence quantification analysis (RQA), as described below. Across subjects and pathways, the median optimal embedding dimension and associated delays used in this study are provided in S1 Appendix Table J. The “*nonlinearTSeries*” package in R (v3.5.2) was used to calculate associated parameters and visualize Takens reconstructions; the full code to implement these procedures as shown throughout this paper is provided in the S1 and S2 Codes.

### Recurrence quantification analysis

We have previously described the application of recurrence quantification analysis (RQA) to elemental time series as applied here [29,48]. RQA was developed to characterize periodic processes in signals from physical and biological systems, and has been extensively applied in diverse fields including geology and climatology, physiology and molecular biology, and neuroscience and psychology, among others (see reviews by Webber *et al*. [49, 50] and Marwan *et al*. [47]). Briefly, the application of RQA involves the construction of a recurrence plot, derived from an attractor reconstructed via Takens lag-embedding method (see above section), whereby a threshold function, *ε*, is successively applied to each lag-embedded point and a matrix is constructed to capture the index value (timing) of any neighboring points within *ε*, with recurrent points represented in black. A process yielding a repeating sequence, e.g. a cycle, will thereby yield the reconstruction of a diagonal line in the recurrence matrix, and the distribution of diagonal lines will reflect the timing and duration of the periodic process. Likewise, during periods when the signal remains relatively stable, such that successive measurements remain within the threshold function *ε*, laminar patterns, e.g. vertical and horizontal lines, or square “boxy” structures, will be apparent in the recurrence plot. Points in phase space that are never revisited will be apparent in the recurrence plot as white space. To facilitate comparison across elemental pathways that may differ in their concentrations and properties, in all RQA analyses conducted here an adaptive threshold value was used such that *ε* yielded a fixed recurrence rate of 10%, as in prior studies that examined RQA features in elemental metabolism [29, 48, 51]. The median lag and embedding dimensions derived during RQA of varying elemental pathways are reported in S1 Appendix Table J.

To quantify cyclical processes in the underlying signal, the distribution of diagonal lines is quantified in RQA to yield measures of determinism, entropy, and mean diagonal length. Determinism quantifies the prevalence of periodic processes as the ratio of cyclical recurrences to all other recurrence points, as defined as $Determinism=\frac{\sum_{{L=l}_{min}}^{N}lP(l)}{\sum_{l=1}^{N}lP(l)}$, where *P*(*l*) defines the histogram of diagonal lines of length *l* derived from a recurrence plot, thus the numerator $\sum_{l=l_{min}}^{N}lP(l)$ reflects all diagonal lines above a minimal length (here, 3 successive measurements), and the denominator $\sum_{l=1}^{N}lP(l)$ reflects all other structures in the recurrence plot. Similarly, mean diagonal length (MDL), which captures the average duration of cyclical processes, can thus be formulated as $MDL=\frac{\sum_{l=l_{min}}^{N}lP(l)}{\sum_{l=l_{min}}^{N}P(l)}$. The variability in cyclical processes is measured through Shannon entropy, formulated as $ENT=-\sum_{l=l_{min}}^{N}p(l)\ln p(l)$, which describes the probabily *p(l)* to find a diagonal line of exactly length l in the recurrence plot; in other words, the complexity or predictability of the system.

RQA was implemented here via the Cross-Recurrence Toolbox v5.16 in Matlab v2016b (MathWorks); example code to recreate recurrence analyses and associated visualizations as shown throughout this paper is provided in the S1 Code. Features derived from RQA analysis of individual traces, i.e. measures of determinism, MDL, and entropy, were used in subsequent statistical analysis in order to test for differences in periodicity at the level of groups, e.g. ALS cases vs. healthy controls.

### Potential energy landscapes

Potential analysis was used to quantify the number of distinct attractor states present in the reconstructed attractor phase space, as applied in previous climatic [12], ecological [21], and microbiological [11] studies of attractor dynamics. This method assumes an underlying stochastic system with a potential energy function following the form:

dz=−U(z)dt+σdW

where *U*(z) is the potential function, z is the time-series of elemental concentrations, *σ* is the standard deviation in concentrations (here, standardized to 1), and dW reflects a Wiener entropy process. The potential function, *U*, is linked to the probability density function of elemental concentration by the Fokker-Planck equation, yielding *U* = $-\frac{\sigma^{2}}{2}\log P_{d}$, where P_d_ is the empirically estimated probability density function of the elemental concentration. Note that this steady-state approximation relies on the assumption that a univariate time series, as elemental concentrations were treated in our analyses, can generally be assumed to meet the assumptions of a gradient system [14].

Probability density functions were estimated with Gaussian density kernels and automated kernel width adjustment with Scott’s method [52]. Potentials were scaled to the noise levels (U/*σ*^2^), following Hirota et al. [21]. As in Lahti et al. [11] local minima and maxima were estimated numerically, with the number of local minima used to quantify the number of distinct attractor states, and the local maxima used to define transitions between states. The number of discrete quasi-stable states was measured for each element, per subject, and then used in subsequent statistical analyses. To visualize state-formation across both time and elemental concentration, potential energy analyses were also conducted in rolling windows, allowing potentials to be assigned to discrete time points, across grids sized at 50 measurements. The “*earlywarnings”* package in the R programming language was used in calculating these functions; full code for the implementation of these analyses and associated visualizations as shown throughout this paper is provided in the S2 Code.

### Statistical analyses

Potential energy analysis and RQA were used to extract, in each individual subject and from each elemental pathway, measures describing the number of quasi-stable states observed, and the determinism, mean diagonal length, and entropy in the elemental exposure profile. Frequency counts of quasi-stable states were analyzed with Poisson regression models to test the effect of ALS diagnosis on the frequency (counts) of stable states throughout development, with covariate adjustments for sex and age at diagnosis. History of tobacco use was included in the model but was not significant. RQA features (determinism, entropy, MDL) were evaluated in general linear models to test for differences in these features between ALS cases and controls, with covariate adjustment for sex and age at diagnosis. For predictive models, both linear and tree-based algorithms were evaluated to test the predictive efficacy of dynamical features. In the linear approach, a least absolute shrinkage selection operator (LASSO) classification model was trained on 40% of the available data (randomly sampled), and tested on 60% of the remaining data. In parallel a tree-based gradient boosting algorithm [53–55] was applied, similarly following a 40/60% random divide of the data for training/testing, with an additional step of 3-fold cross-validation applied in the training step. The data used in both predictive models included only features derived from potential analysis and RQA for each element, e.g. frequency of quasi-stable states, and determinism, entropy, and MDL. Following the method of Mason and Graham [56], we used rank-based tests to evaluate the hypothesis that ROC curves yielded better performance than would be expected by chance. All statistical analyses were implemented in the R programming language using packages *glm2*, *glmnet*, and *Xgboost* for generalized linear models, predictive LASSO, and tree-based gradient boosting, respectively. All statistical tests were conducted against an alpha of 0.05 for purposes of determining statistical significance; p-values are reported both in raw, unadjusted form, and following FDR adjustment for multiple comparisons.

## Supporting information

S1 Appendix Supporting information for this manuscript including Figures, Tables, Captions, Procedures and References. (DOCX)

S1 Code Example code for recurrence quantification analysis. (M)

S2 Code Example code for potential energy landscapes. (R)

S1 Movie Potential energy landscape shows multiple attractor wells separated in a control subject. (MP4)

S2 Movie Potential energy landscape shows a single well attractor system in an ALS case. (MP4)
