# Supplementary material for: Dysregulated biodynamics in metabolic attractor systems precede the emergence of amyotrophic lateral sclerosis
Source: PLoS Comput Biol. 2020 Apr 15;16(4):e1007773. doi: 10.1371/journal.pcbi.1007773 (PMC7159190; doi:10.1371/journal.pcbi.1007773)
Supplement: S1 Appendix — (DOCX) [file pcbi.1007773.s001.docx]

S1 Appendix for

**Dysregulated biodynamics in metabolic attractor systems precedes the emergence of amyotrophic lateral sclerosis**

Paul Curtin PhD^1,†*^, Christine Austin PhD^1,†^, Austen Curtin PhD^1,†^, Chris Gennings PhD^1,†^, Claudia Figueroa-Romero, PhD^2^, Kristen A. Mikhail, MPH^2^, Tatiana M. Botero, DDS, MS^3^, Stephen A. Goutman, MD^2^, Eva L. Feldman, MD, PhD^2^ , Manish Arora BDS., PhD^1,†*^

Affiliations:

^1^Department of Environmental Medicine and Public Health, Icahn School of Medicine at Mount Sinai, One Gustave L Levy Place, Box 1057, New York, NY 10029, USA.

^2^Department of Neurology, University of Michigan, Ann Arbor, MI 48109 USA

^3^Department of Cariology, Restorative Sciences and Endodontics, School of Dentistry University of Michigan, Ann Arbor, MI 48109 USA

^†^Emergent Dynamical Systems (EDS) group; authors contributed equally *Correspondence: [paul.curtin@mssm.edu](mailto:paul.curtin@mssm.edu) (P.C.), [manish.arora@mssm.edu](mailto:manish.arora@mssm.edu) (M.A.)

**This PDF file includes:**

Figures A-E

Tables A-J

Captions for Movies 1 to 2

Supplemental Procedures

Supplemental References

**Other Supplementary Materials for this manuscript include the following:**

S1 Code. Example Code for Potential Analysis.

S2 Code. Example Code for RQA Analysis.

S1 Movie

S2 Movie


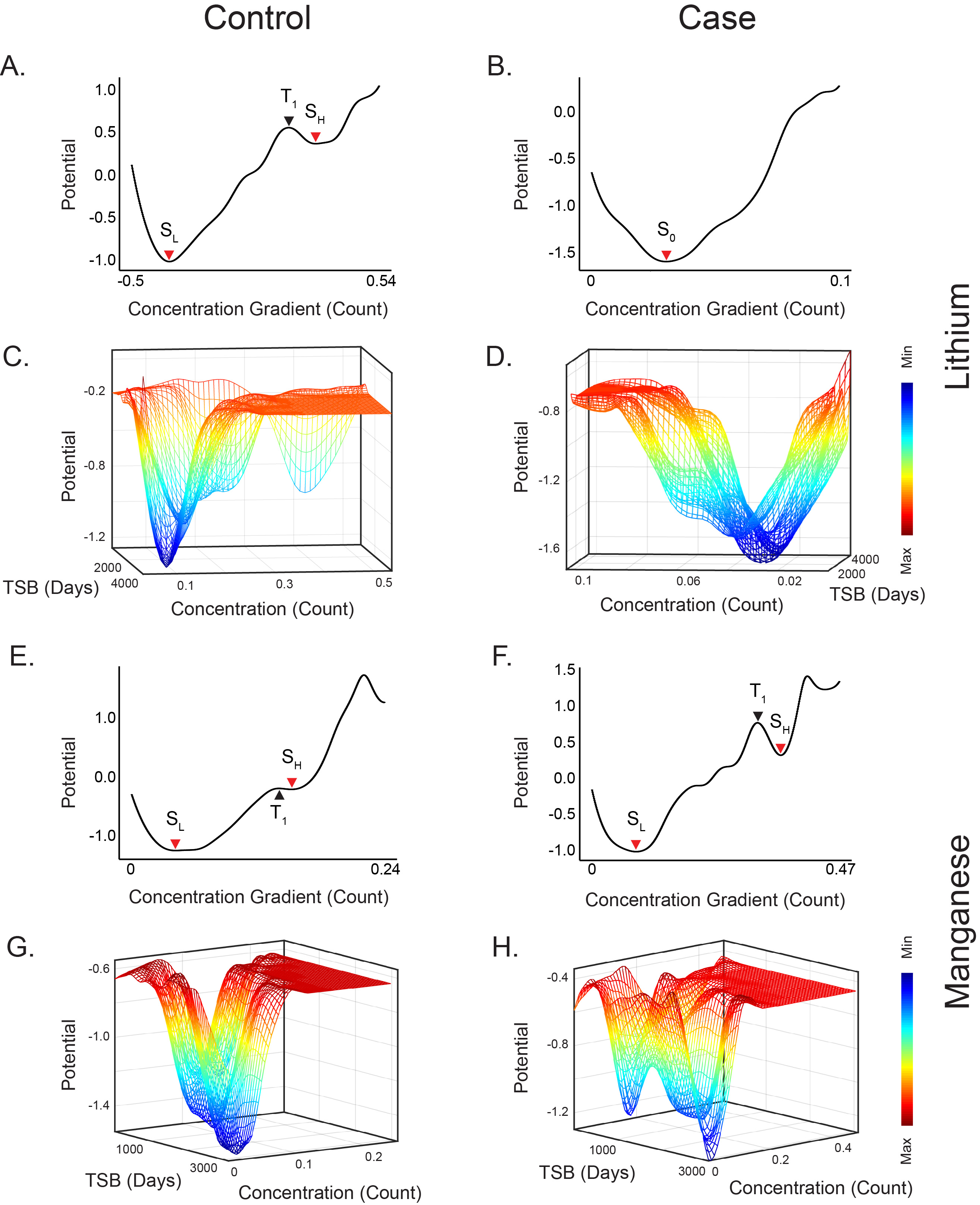


**Figure A. Potential energy profiles in controls and ALS cases.** **(A, B)** Lithium potential energy profiles in a healthy control (A) and ALS case (B). Note, in controls that lithium transitions between a low oscillating state (S_L_) and a high oscillating state (S_H_). **(C)** Potential energy landscape of lithium metabolism in a healthy control shows a multi-well attractor system. **(D)** Lithium landscape in an ALS case with a single attractor basin that fails to differentiate as observed in healthy controls. **(E, F)** Manganese concentration profiles in a healthy control (E) and ALS case (F). **(G, H)** Manganese potential energy profiles show a bistable attractor system with two wells in controls (G), and this remains unchanged in ALS cases (H).


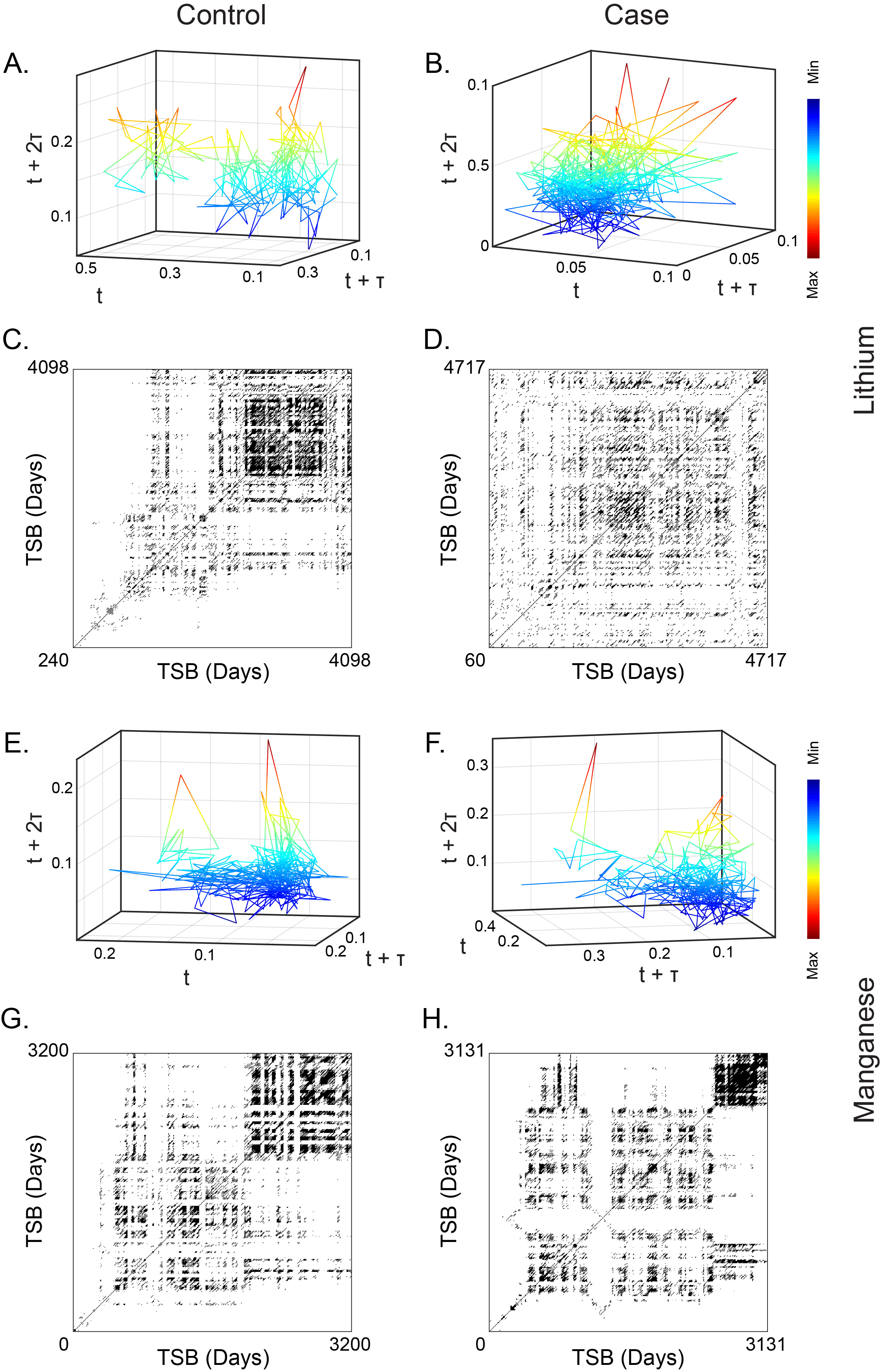


**Figure B. Phase space embedding of elemental concentration profiles.** Plots show phase portraits derived from Takens embeddings of elemental concentration profiles corresponding to potential energy landscapes shown in Figure A. **(A)** Lithium phase portrait from a healthy control subject suggests a bistable attractor system. Axes

are derived from lag embedding of lithium measurements and are therefore in units of count (Li). **(B)** Unitary lithium attractor system observed in an ALS case. **(C)** Recurrence plot emphasizing the change in dynamic structure in (A). (D) Recurrence plot derived from (B) capturing the loss of dynamic structure in ALS cases. **(E)** Bistable manganese attractor system observed in a control subject, and this remains unchanged in ALS cases **(F)**. **(G, H)** Recurrence plot structure of (G) in a control subject remains similar in an ALS case (H).


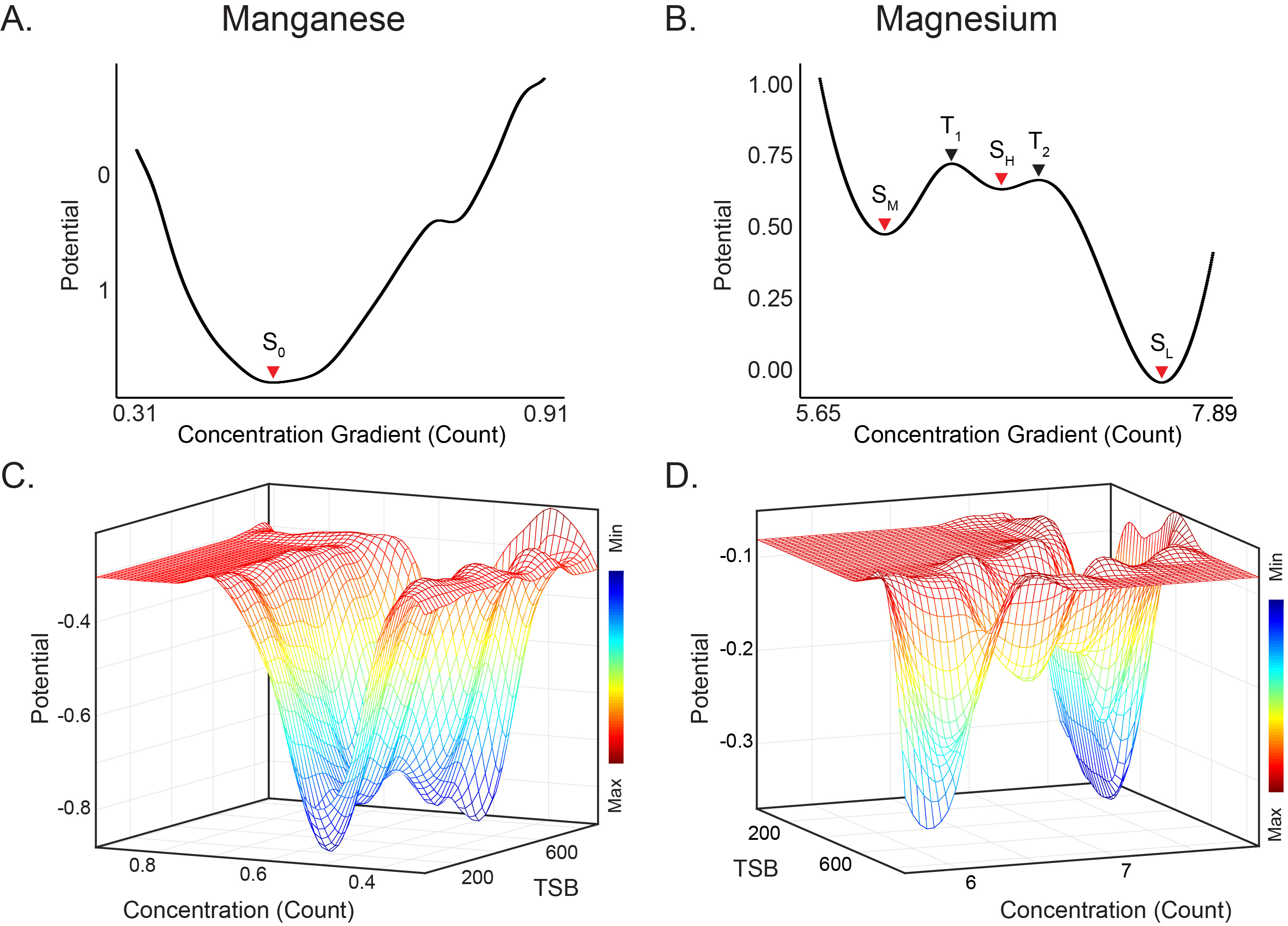


**Figure C.** **Potential energy landscapes in wild-type mice.** **(A, B)** show example potential energy profiles extracted from manganese (A) and magnesium (B) traces in a control rodent. **(C, D)** show corresponding potential energies plotted with developmental timing and elemental intensity, emphasizing the formation of a prolonged stable state in manganese, and the characteristic transitions between discrete states observed in magnesium.


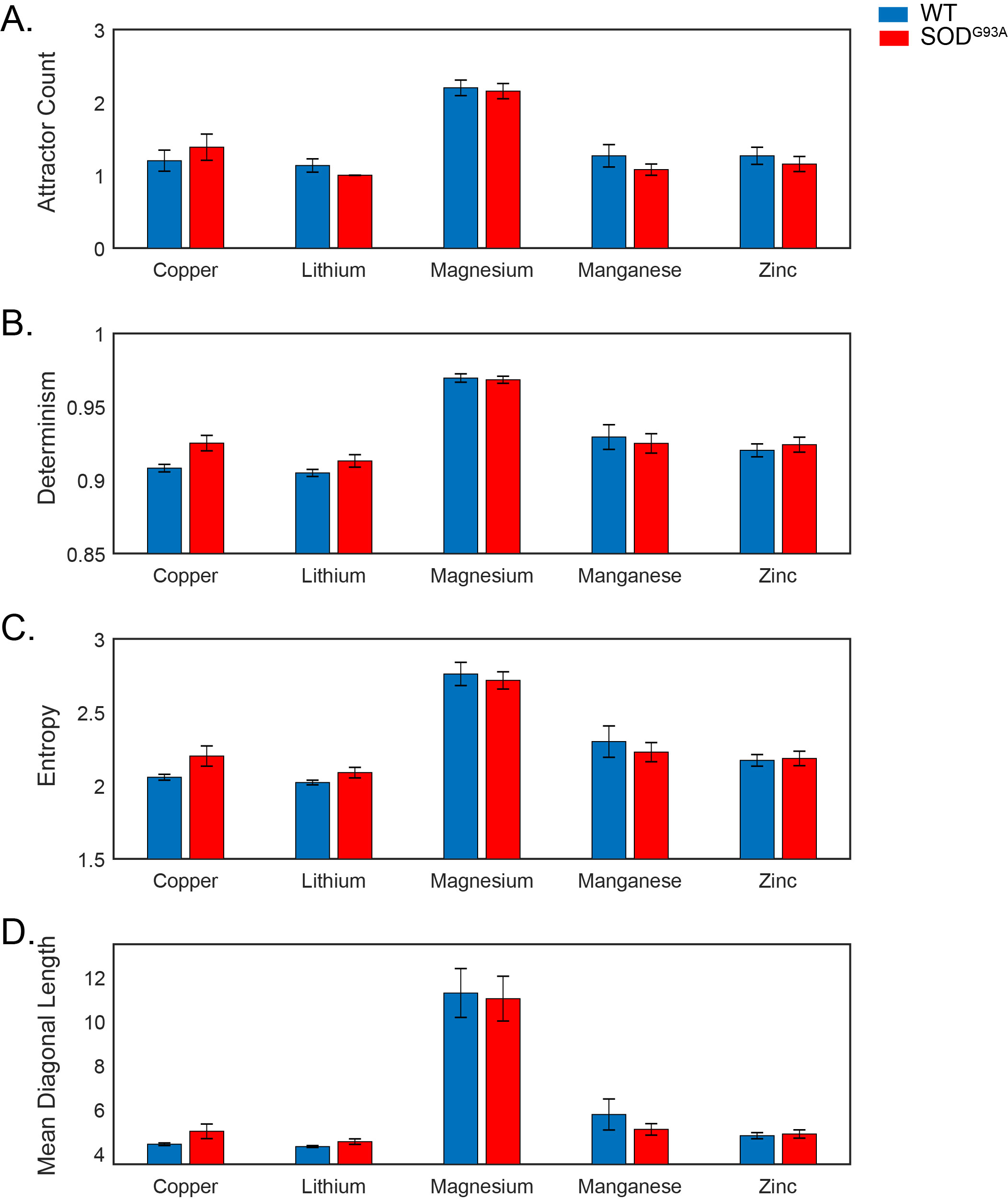


**Figure D. Distribution of elemental attractor states and recurrence features in WT mice controls and SOD1^G93A^ mutant mice.** In **(A)**, the mean (± SEM) number of quasi-stable states identified in WT mice controls (blue bars) and SOD1^G93A^ mutant mice (red bars) are shown in varying elemental pathways. **(B**, **C**, **D)** show RQA-derived mean (± SEM) of Determinism (B), Entropy (C), or Mean Diagonal Length (D) for SOD1^G93A^ mutant mice (red bars) and WT controls (blue).

**Overview of Tooth Development**


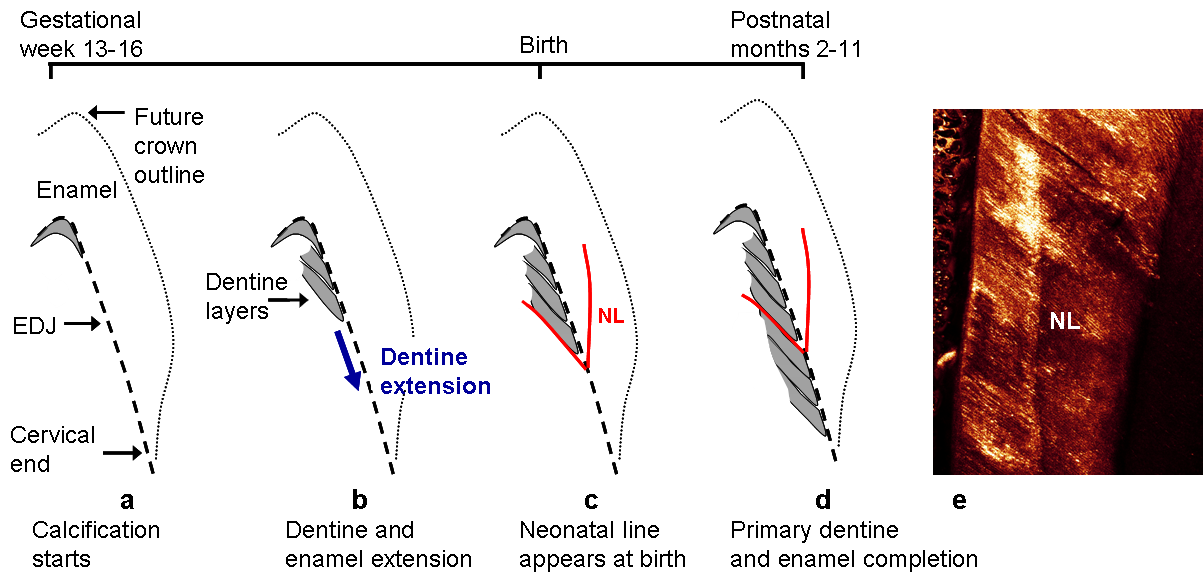


**Figure E. Schematic of tooth development.** **(A)** Earliest deposition of dentine (grey area) at the dentine horn tip. **(B)** Continued extension of dentine (and enamel) along the enamel-dentine junction (EDJ) towards tooth cervix. **(C)** In all deciduous teeth and permanent first molars, the neonatal line (NL), a histological feature is formed at the time of birth. **(D)** Completion of enamel and dentine formation in deciduous teeth at 2 to 11 postnatal months, depending on tooth type. Dentine formation then extends to the tooth root (not shown). **(E)** Confocal laser scanning micrograph of NL in enamel. *Reprinted with permission from Arora et al. Environ. Sci. Technol., 2012, 46 (9), pp 5118–5125. Copyright 2012 American Chemical Society.*

**Figure F.** **Overview of Laboratory Protocol for Tooth Analysis.** **(A)** Tooth is sectioned along the longitudinal axis and the laser is scanned from the dentine horn in the cusp tip to the root tip indicated by the dashed black line. **(B)** Electron micrograph showing ablated dentine after laser scanning. **(C)** The ablated dentine material is transferred to an inductively coupled plasma-mass spectrometer where a quadrupole separated the ions based on mass-to-charge ratio and an ion detector provides ion counts as a measure of intensity.

**C**

**A**

**B**


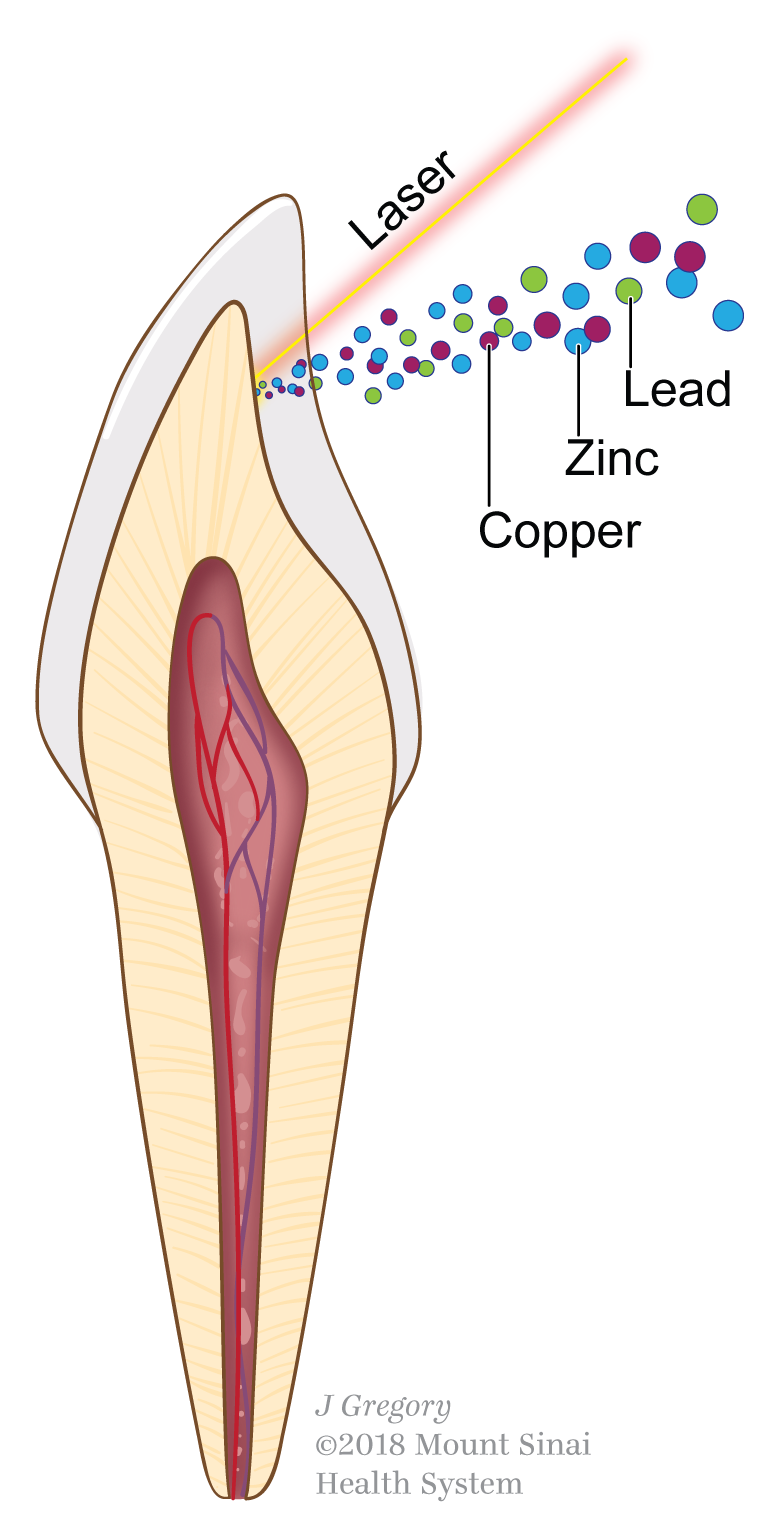

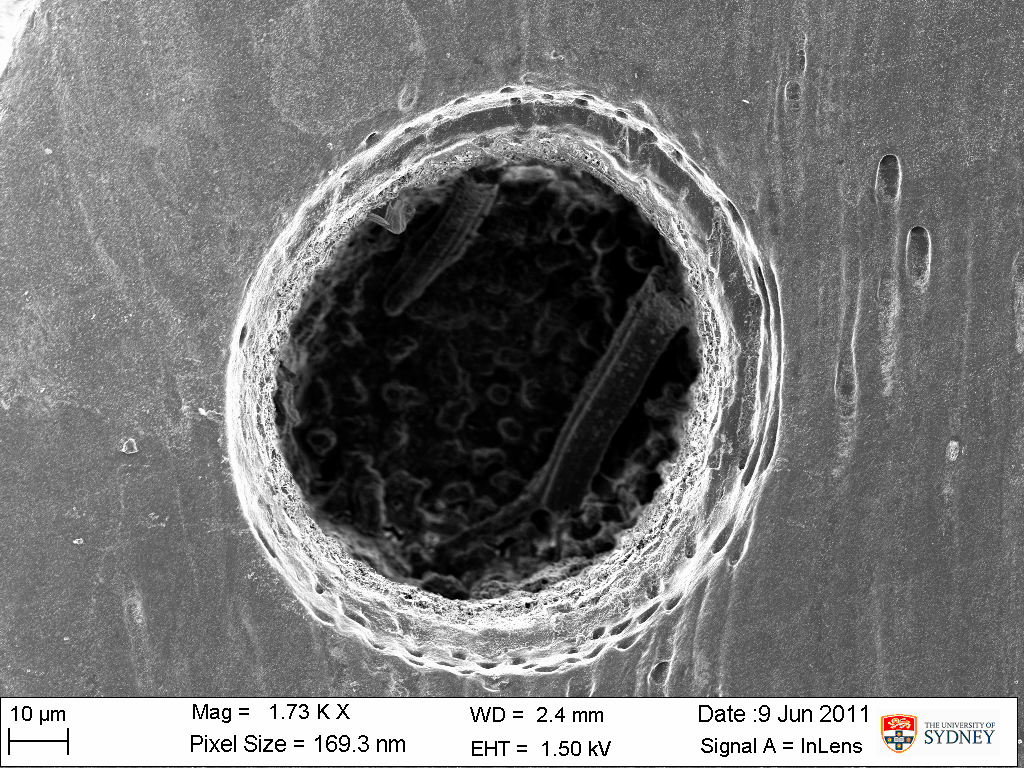


Ion Detector

Ion Detector

**Table A.** Case *vs.* control differences in elemental attractor characteristics.

|  | Case Status | Median | Mean | SEM |
| --- | --- | --- | --- | --- |
| **Copper** | Control | 2 | 2.9 | 0.5 |
|  | ALS | 1 | 1 | 0 |
| **Lithium** | Control | 2 | 2.5 | 0.4 |
|  | ALS | 1 | 1.7 | 0.2 |
| **Magnesium** | Control | 1 | 1 | 0 |
|  | ALS | 1 | 1 | 0 |
| **Manganese** | Control | 2 | 2.5 | 0.4 |
|  | ALS | 2 | 2.4 | 0.2 |
| **Zinc** | Control | 1 | 1 | 0 |
|  | ALS | 1 | 1 | 0 |

**Table B.** Demographics of study participants

| **Characteristics** | | | **ALS group (n=36)** | **Control group (n=31)** | ***p* value** | |
| --- | --- | --- | --- | --- | --- | --- |
| Age (years)^(a,b,c)^ |  | 63.0, 62.11 ± 1.757, (27-87) | | 57.5, 55.45 ± 2.142 (25-74) | | 0.0180^(f)^ |
| Sex | Male | 17 (47.22%) | | 16 (51.61%) | | 0.8083^(g)^ |
|  | Female | 19(52.78%) | | 15 (48.39%) | | - |
| Onset | Bulbar | 13 (36.11%) | | - | | - |
|  | Limb | 23 (63.89%) | | - | | - |
| Family history/known causative gene | Yes^(d)^ | 4 (11.11%) | | - | | - |
|  | No^(e)^ | 32 (88.89%) | | - | | - |
| Ethnicity | Caucasian | 35 (97.22%) | | 26 (83.87%) | | 0.0472^(h)^ |
|  | AA | - | | 4(12.90%) | | - |
|  | Other | - | | 1(3.22%) | | - |
|  | NA | 1(2.78%) | | 0 | |  |
| Tobacco | Yes | 18 (50.00%) | | 24 (77.42%) | | 0.0035^(g)^ |
|  | No | 18 (50.00%) | | 4 (12.90%) | | - |
|  | NA | - | | 3 (9.68%) | | - |
| a) median; (b) mean ± standard error; (c) range; (d) familial ALS was determined by known family history or genetic alterations for *SOD1* or *C9orf 72*; (e) one adopted participant was classified as sporadic ALS as it was negative for abnormal C9orf72 expansions; (f) Student’s t-test; (g) Fisher's exact test: male and female for sex, Yes and No answers for smoking; (h) Chi-Square Test: Caucasian, AA, and other for ethnicity; AA, African American; NA, Data not available | | | | | | |

**Table C.** Analysis of stable state dynamics.

| Element | β | Lower  Bound | Upper  Bound | P | FDR |
| --- | --- | --- | --- | --- | --- |
| Cu | -1.08 | -1.47 | -0.69 | 0.00 | 0.00 |
| Li | -0.37 | -0.71 | -0.04 | 0.03 | 0.07 |
| Mg | 0.00 | -0.48 | 0.48 | 1.00 | 1.00 |
| Mn | -0.07 | -0.38 | 0.23 | 0.64 | 1.00 |
| Zn | 0.00 | -0.48 | 0.48 | 1.00 | 1.00 |

*β* refers to the estimated regression parameter associated with ALS diagnosis in a model predicting the number of stable states formed in a given elemental pathway, with adjustment for sex and age at diagnosis. Lower and upper bounds provide 95% confidence intervals on *β* parameters, *P* provides associated *P* values, and *FDR* provides False Discovery Rate adjusted *P*-values.

**Table D.** Results of human recurrence analysis.

| Measure | Element | β | Lower  Bound | Upper  Bound | P | FDR |
| --- | --- | --- | --- | --- | --- | --- |
| Determinism | **Cu** | -0.01 | -0.02 | 0.00 | 0.04 | 0.06 |
|  | **Li** | 0.00 | -0.01 | 0.01 | 0.58 | 0.79 |
|  | **Mg** | -0.02 | -0.03 | -0.01 | 0.00 | 0.00 |
|  | **Mn** | -0.01 | -0.02 | 0.00 | 0.02 | 0.04 |
|  | **Zn** | 0.00 | -0.01 | 0.01 | 0.68 | 0.79 |
| Entropy | **Cu** | -0.15 | -0.27 | -0.02 | 0.03 | 0.05 |
|  | **Li** | 0.00 | -0.07 | 0.06 | 0.96 | 0.96 |
|  | **Mg** | -0.21 | -0.31 | -0.11 | 0.00 | 0.00 |
|  | **Mn** | -0.18 | -0.30 | -0.06 | 0.00 | 0.01 |
|  | **Zn** | 0.03 | -0.09 | 0.14 | 0.64 | 0.79 |
| Mean Diagonal Length | **Cu** | -0.76 | -1.42 | -0.11 | 0.02 | 0.05 |
|  | **Li** | -0.03 | -0.23 | 0.18 | 0.81 | 0.86 |
|  | **Mg** | -0.95 | -1.37 | -0.52 | 0.00 | 0.00 |
|  | **Mn** | -1.00 | -1.67 | -0.33 | 0.00 | 0.01 |
|  | **Zn** | 0.11 | -0.39 | 0.62 | 0.66 | 0.79 |

*β* refers to the estimated regression parameter in a model predicting the mean difference in a given recurrence parameter (Determinism, Mean Diagonal Length, Entropy) between ALS cases and controls in a given elemental pathway, with adjustment for sex and age at diagnosis. Lower and upper bounds provide 95% confidence intervals on *β* parameters, *P* provides associated *P* values, and *FDR* provides False Discovery Rate adjusted *P*-values.

**Table E.** Attractor dynamics in rodent animal models.

|  | **Genotype** | **Median Attractor Count** | **Mean** | **SEM** |
| --- | --- | --- | --- | --- |
| **Copper** | Wild-type | 1 | 1.20 | 0.14 |
|  | SOD1^G93A^ | 1 | 1.38 | 0.18 |
| **Lithium** | Wild-type | 1 | 1.13 | 0.09 |
|  | SOD1^G93A^ | 1 | 1 | 0 |
| **Magnesium** | Wild-type | 2 | 2.20 | 0.11 |
|  | SOD1^G93A^ | 2 | 2.15 | 0.10 |
| **Manganese** | Wild-type | 1 | 1.27 | 0.15 |
|  | SOD1^G93A^ | 1 | 1.08 | 0.08 |
| **Zinc** | Wild-type | 1 | 1.27 | 0.12 |
|  | SOD1^G93A^ | 1 | 1.15 | 0.10 |

**Table F.** Results of rodent model recurrence analysis.

| Measure | Element | β | Lower  Bound | Upper  Bound | P | FDR |
| --- | --- | --- | --- | --- | --- | --- |
| Determinism | **Cu** | 0.02 | 0.01 | 0.03 | 0.00 | 0.01 |
|  | **Li** | 0.01 | 0.00 | 0.02 | 0.04 | 0.14 |
|  | **Mg** | 0.00 | -0.01 | 0.00 | 0.59 | 0.74 |
|  | **Mn** | -0.01 | -0.02 | 0.01 | 0.32 | 0.53 |
|  | **Zn** | 0.00 | -0.01 | 0.01 | 0.55 | 0.74 |
| Entropy | **Cu** | 0.14 | 0.03 | 0.26 | 0.02 | 0.13 |
|  | **Li** | 0.06 | 0.00 | 0.13 | 0.06 | 0.14 |
|  | **Mg** | -0.06 | -0.22 | 0.10 | 0.48 | 0.73 |
|  | **Mn** | -0.08 | -0.23 | 0.06 | 0.27 | 0.50 |
|  | **Zn** | 0.00 | -0.09 | 0.10 | 0.93 | 0.93 |
| Mean Diagonal Length | **Cu** | 0.58 | 0.02 | 1.15 | 0.04 | 0.14 |
|  | **Li** | 0.21 | -0.01 | 0.43 | 0.06 | 0.14 |
|  | **Mg** | -0.36 | -2.83 | 2.10 | 0.77 | 0.84 |
|  | **Mn** | -0.73 | -1.79 | 0.34 | 0.18 | 0.39 |
|  | **Zn** | 0.05 | -0.30 | 0.40 | 0.79 | 0.84 |

*β* refers to the estimated regression parameter in a model predicting the mean difference in a given recurrence parameter (Determinism, Mean Diagonal Length, Entropy) between mutant SOD1^G93A^ and controls in a given elemental pathway, with adjustment for age and sex. Lower and upper bounds provide 95% confidence intervals on *β* parameters, and *P* provides associated *P* values.

**Table G.** Parameter estimates from LASSO model.

| Feature | LASSO Parameter  Estimate |
| --- | --- |
| Number of Copper States | 0.00 |
| Number of Lithium States | -1.27 |
| Number of Magnesium States | 0.00 |
| Number of Manganese States | -0.06 |
| Number of Zinc States | 0.00 |
| Copper Determinism | -267.41 |
| Lithium Determinism | 23.70 |
| Magnesium Determinism | -35.61 |
| Manganese Determinism | 48.30 |
| Zinc Determinism | -96.92 |
| Copper Entropy | 43.44 |
| Lithium Entropy | 63.10 |
| Magnesium Entropy | 123.90 |
| Manganese Entropy | -68.19 |
| Zinc Entropy | 61.47 |
| Copper MDL | -3.64 |
| Lithium MDL | -17.60 |
| Magnesium MDL | -28.80 |
| Manganese MDL | 8.37 |
| Zinc MDL | -11.68 |

**Table H.** Feature Importance in gradient boosting model.

| Feature | Gain |
| --- | --- |
| Magnesium Entropy | 0.30 |
| Manganese Determinism | 0.16 |
| Copper Determinism | 0.14 |
| Manganese MDL | 0.12 |
| Magnesium Determinism | 0.07 |
| Lithium Determinism | 0.06 |
| Zinc Entropy | 0.04 |
| Lithium Entropy | 0.03 |
| Magnesium MDL | 0.02 |
| Manganese Entropy | 0.02 |
| Copper MDL | 0.02 |
| Copper Entropy | 0.01 |
| Number of Lithium States | 0.01 |
| Number of Copper States | -- |
| Number of Magnesium States | -- |
| Number of Manganese States | -- |
| Number of Zinc States | -- |
| Zinc Determinism | -- |
| Lithium MDL | -- |
| Zinc MDL | -- |

**Table I.** LA-ICP-MS operating conditions.

| **NWR-193 Laser Conditions** | | **Agilent 8800 ICP-MS Conditions** | |
| --- | --- | --- | --- |
| Wavelength (nm) | 193 | RF power (W) | 1350 |
| Helium carrier flow (L min^-1^) | 0.8 | Argon carrier flow (L min^-1^) | 0.6 |
| Fluence (J cm^-1^) | 5.0 | Plasma gas flow (L min^-1^) | 15 |
| Repetition rate (Hz) | 10 | Sample Depth (mm) | 4.0 |
| Spot size (μm) | 35 | Scan mode | Peak hopping |
| Scan speed (μm s^-1^) | 35 | Integration time (ms) | 50 – 55 |

**Table J.** Parameters for RQA analysis.

| Metal | N | Median Embedding Dimensions | Lag | Threshold  (Fixed) |
| --- | --- | --- | --- | --- |
| Copper | 67 | 6 | 1 | 0.1 |
| Lithium | 67 | 6 | 1 | 0.1 |
| Magnesium | 67 | 5 | 1 | 0.1 |
| Manganese | 67 | 6 | 1 | 0.1 |
| Zinc | 67 | 6 | 1 | 0.1 |

**Supplemental Movie Legends**

**S1 Movie.**

Potential energy landscape shows multiple attractor wells separated in a control subject.

**S2 Movie.**

Potential energy landscape shows a single well attractor system in an ALS case.

**Supplemental Procedures**

**Metal analysis of teeth**

Metal concentrations were determined by laser ablation-inductively coupled plasma-mass spectrometry (LA-ICP-MS) (Figure F) (1-3). Briefly, we used a New Wave Research NWR-193 (ESI, USA) laser ablation unit equipped with a 193 nm ArF excimer laser connected to an Agilent Technologies 8800 triple-quad ICP-MS (Agilent Technologies). The laser was scanned in dentine from the dentine horn tip towards the tooth root tip and surface contamination was removed using a pre-ablation scan. Data were analyzed as metal (metal studied) to calcium (internal standard) ratios to control for any variations in mineral content within a tooth and between samples. Each tooth was sampled, on average, at over 500 locations. Operating parameters for LA-ICP-MS are shown in Supplementary Table S9. Quality assurance and quality control protocols include the analysis of NIST certified standards before and after every tooth. Helium was the carrier gas from the laser ablation cell and it was mixed with argon before entering the ICP-MS via a Y-piece. To ensure accuracy and precision, sensitivity (maximum analyte ion counts), oxide formation (^232^Th^16^O^+^/^232^Th^+^, < 0.3%), and fractionation (^232^Th^+^/^238^U^+^, 100 ± 5%) were monitored daily using NIST SRM 612 (trace elements in glass). In addition, measured concentrations are compared to certified levels and values outside +/- 20% trigger a reanalysis. Finally, a sample tooth is reanalyzed with every run to ensure a matrix-matched standard and within run and between run comparability is constantly monitored. Our analytical protocols have been validated previously (4, 5).

**Validation of Tooth Elemental Biomarkers**

***Tooth metal concentrations vs levels in environmental samples and blood***

Metal concentrations in deciduous and permanent teeth have been validated against levels in environmental samples and in blood in both human and animal studies. Here, we provide a summary of the validation studies and refer reader to primary articles reporting those studies.

In two studies, we compared lead and manganese levels in teeth from rats given controlled doses of the metals separately. In both studies we saw a clear and strong relationship with exposure level and concentration in teeth. In the study on manganese exposure, we observed a positive association between tooth manganese and manganese levels in blood (Spearman's rho 0.69, p<0.01), brain (rho 0.59, p<0.05) and bone (rho 0.69, p<0.01) in animals with lifelong exposure.(6) In the study on lead exposure, we found the correlation between lead levels in teeth with bones, brain, kidney and liver to range from 0.82 to 0.91 (p<0.001) (7).

Several human studies have also been undertaken on the validation and application of elemental distribution in teeth. The earliest studies by Needleman and colleagues showed that lead levels in teeth were higher among children living in high lead environments.(8-11) Work by Gulson and colleagues showed that teeth contain the imprints of lead released from bone and may be used to identify the source of exposure (12, 13). We validated tooth metals levels against levels in environmental samples, such as soil and house dust, and also in longitudinal blood samples (4, 14, 15).

***Tooth metal concentrations vs population demographics and health outcomes***

An important validation of any exposure biomarker is its ability to reproduce known differences in population exposures and also its relationship with health outcomes. We have shown that tooth metal levels are related to sources of exposure (proximity to metal containing pesticide use, for example (4) or differences between racial/ethnic groups (16).

We have also shown that our laboratory method provides metal distribution data that reveals dynamic properties of metal metabolism which can uncover risk of neurodevelopmental disorders such as autism spectrum disorder (2, 17).

**Supplemental References**

1. Andra SS, Austin C, Arora M. The tooth exposome in children's health research. Current opinion in pediatrics. 2016;28(2):221-7.

2. Arora M, Reichenberg A, Willfors C, Austin C, Gennings C, Berggren S, et al. Fetal and postnatal metal dysregulation in autism. Nat Commun. 2017;8:15493.

3. Modabbernia A, Velthorst E, Gennings C, De Haan L, Austin C, Sutterland A, et al. Early-life metal exposure and schizophrenia: A proof-of-concept study using novel tooth-matrix biomarkers. Eur Psychiatry. 2016;36:1-6.

4. Arora M, Bradman A, Austin C, Vedar M, Holland N, Eskenazi B, et al. Determining fetal manganese exposure from mantle dentine of deciduous teeth. Environ Sci Technol. 2012;46(9):5118-25.

5. Austin C, Smith TM, Bradman A, Hinde K, Joannes-Boyau R, Bishop D, et al. Barium distributions in teeth reveal early-life dietary transitions in primates. Nature. 2013;498(7453):216-9.

6. Austin C, Richardson C, Smith D, Arora M. Tooth manganese as a biomarker of exposure and body burden in rats. Environmental research. 2017;155:373-9.

7. Arora M, Hare D. Tooth lead levels as an estimate of lead body burden in rats following pre- and neonatal exposure RSC Advances. 2015(5).

8. Needleman HL, Davidson I, Sewell EM, Shapiro IM. Subclinical lead exposure in philadelphia schoolchildren. Identification by dentine lead analysis. N Engl J Med. 1974;290(5):245-8.

9. Needleman HL, Gatsonis CA. Low-level lead exposure and the IQ of children. A meta-analysis of modern studies. JAMA. 1990;263(5):673-8.

10. Needleman HL, Schell A, Bellinger D, Leviton A, Allred EN. The long-term effects of exposure to low doses of lead in childhood. An 11-year follow-up report. N Engl J Med. 1990;322(2):83-8.

11. Needleman HL, Shapiro IM. Dentine lead levels in asymptomatic Philadelphia school children: subclinical exposure in high and low risk groups. Environmental health perspectives. 1974;7:27-31.

12. Gulson B, Taylor A, Eisman J. Bone remodeling during pregnancy and post-partum assessed by metal lead levels and isotopic concentrations. Bone. 2016;89:40-51.

13. Gulson BL, Gillings BR. Lead exchange in teeth and bone--a pilot study using stable lead isotopes. Environmental health perspectives. 1997;105(8):820-4.

14. Arora M, Austin C, Sarrafpour B, Hernandez-Avila M, Hu H, Wright RO, et al. Determining prenatal, early childhood and cumulative long-term lead exposure using micro-spatial deciduous dentine levels. PLoS One. 2014;9(5):e97805.

15. Johnston JE, Franklin M, Roh H, Austin C, Arora M. Lead and Arsenic in Shed Deciduous Teeth of Children Living Near a Lead-Acid Battery Smelter. Environ Sci Technol. 2019.

16. Cassidy-Bushrow AE, Sitarik AR, Havstad S, Park SK, Bielak LF, Austin C, et al. Burden of higher lead exposure in African-Americans starts in utero and persists into childhood. Environment international. 2017;108:221-7.

17. Curtin P, Austin C, Curtin A, Gennings C, Arora M, Tammimies K, et al. Dynamical features in fetal and postnatal zinc-copper metabolic cycles predict the emergence of autism spectrum disorder. Sci Adv. 2018;4(5):eaat1293.
